# Rapid susceptibility profiling of carbapenem-resistant *Klebsiella pneumoniae*

**DOI:** 10.1038/s41598-017-02009-3

**Published:** 2017-05-15

**Authors:** K. T. Mulroney, J. M. Hall, X. Huang, E. Turnbull, N. M. Bzdyl, A. Chakera, U. Naseer, E. M. Corea, M. J. Ellington, K. L. Hopkins, A. L. Wester, O. Ekelund, N. Woodford, T. J. J. Inglis

**Affiliations:** 1 Harry Perkins Institute of Medical Research, School of Medicine, Faculty of Health and Medical Sciences, the University of Western Australia, Nedlands, Western Australia Australia; 2 Marshall Centre, School of Biomedical Sciences, Faculty of Health and Medical Sciences, the University of Western Australia, Nedlands, Western Australia Australia; 3 Department of Microbiology, PathWest Laboratory Medicine, WA Nedlands, Australia; 4 Norwegian Institute of Public Health, Oslo, Norway; 5 Department of Microbiology, University of Colombo, Kynsey Road, Colombo, Sri Lanka; 6 Antimicrobial Resistance and Healthcare Associated Infections (AMRHAI) Reference Unit, National Infection Service, Public Health England, London, NW9 5EQ UK; 7 Department of Clinical Microbiology and EUCAST Development Laboratory, Region Kronoberg, Växjö, Sweden; 8 Division of Pathology and Laboratory Medicine, School of Medicine, Faculty of Health and Medical Sciences, the University of Western Australia, Nedlands, Western Australia Australia

## Abstract

The expanding global distribution of multi-resistant *Klebsiella pneumoniae* demands faster antimicrobial susceptibility testing (AST) to guide antibiotic treatment. Current ASTs rely on time-consuming differentiation of resistance and susceptibility after initial isolation of bacteria from a clinical specimen. Here we describe a flow cytometry workflow to determine carbapenem susceptibility from bacterial cell characteristics in an international *K. pneumoniae* isolate collection (n = 48), with a range of carbapenemases. Our flow cytometry-assisted susceptibility test (FAST) method combines rapid qualitative susceptible/non-susceptible classification and quantitative MIC measurement in a single process completed shortly after receipt of a primary isolate (54 and 158 minutes respectively). The qualitative FAST results and FAST-derived MIC (MIC_FAST_) correspond closely with broth microdilution MIC (MIC_BMD_, Matthew’s correlation coefficient 0.887), align with the international AST standard (ISO 200776-1; 2006) and could be used for rapid determination of antimicrobial susceptibility in a wider range of Gram negative and Gram positive bacteria.

## Introduction

The O’Neill Review on Antimicrobial Resistance (AMR) estimated that 700,000 people die from infections due to resistant organisms every year, and by 2050 AMR will surpass cancer as a cause of death^1^. The World Health Organization (WHO) recognises AMR as a serious threat to global health^2^, and singled out the emergence of carbapenem-resistant *Klebsiella* species as the leading priority in its first global report on AMR in 2014^3^. *Klebsiella* species are the most prominent carbapenem-resistant Enterobacteriaceae (CRE) and cause an excess hospital mortality of 27% in patients with septicaemia and pneumonia^4^. The Indian Ocean Rim region has become one of the main foci of emerging carbapenemases, and has seen successive waves of diverse forms of carbapenem-resistant *K. pneumoniae* ^5, 6^.

Faster antimicrobial susceptibility test (AST) methods are an essential component of the multi-faceted measures needed to reduce inappropriate antibiotic use and combat the rise of AMR^1, 7^. Detection of multidrug-resistant bacteria currently relies on primary isolation followed by largely culture-dependent AST procedures, delaying the commencement of targeted treatment and infection prevention and control measures by 24–72 hours. Inappropriate, broad-spectrum antimicrobials are used in the absence of empirical laboratory results, prompting the search for faster methods^5^. Current rapid non-culture-based screening methods such as mechanism-specific PCR and the widely used Carba-NP test, can be unreliable^8^, which diminishes their value for predicting carbapenem susceptibility and thus their utility for the prescribing physician. MIC determination by broth microdilution is the internationally recognized standard for AST (ISO 200776-1, 2006)^9^. A categoric classification (susceptible, intermediate or resistant – SIR, or susceptible/non-susceptible – S/NS) can be made by comparing the MICs to species-specific breakpoints issued either by the European Committee on Antimicrobial Susceptibility Testing (EUCAST) or the Clinical & Laboratory Standards Institute (CLSI). All methods for antimicrobial susceptibility testing must be validated against broth microdilution before introduction into clinical practice^10^.

Flow cytometry has long been considered a candidate method for delivering rapid AST^11^. Early flow cytometry analyses of bacteria were limited by their low resolution to studies of cellular aggregation, but the introduction of bacterial viability dyes, improved flow cytometer resolution, and increased sophistication of multi-parameter analysis has prompted renewed attempts to establish a method for flow-assisted antimicrobial susceptibility analysis^12–16^. These studies produced a catalogue of complex interactions between membrane-permeable dyes and bacteria during sub-lethal damage, but the resolution limits of the best (previously) available hydrodynamic flow cytometers still constrained Flow cytometer-Assisted Susceptibility Test (FAST) methods^17, 18^ with little progress in making FAST methods widely accessible.

Here we report an acoustic flow cytometry (AFC) method capable of rapidly determining carbapenem MICs and assigning susceptibility categories. Furthermore, we compared the method against broth microdilution in a blinded, prospective validation using a collection of carbapenem-susceptible and carbapenem-resistant *K. pneumoniae* and *K. oxytoca* isolates, including internationally dominant ESBL- and carbapenemase-producing isolates.

## Materials and Methods

### Bacterial isolates

**Figure 1:**
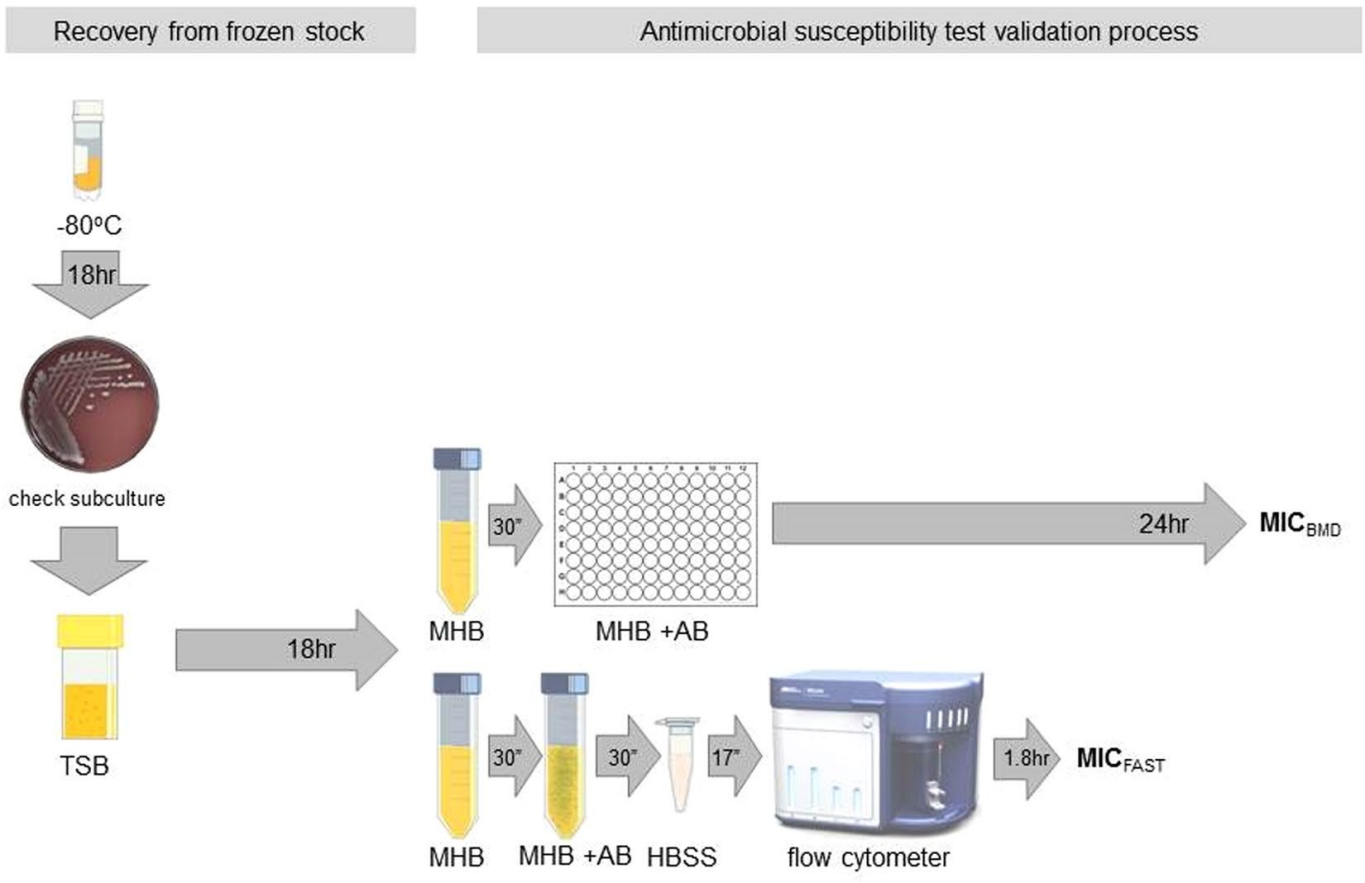
Schematic diagram of flow cytometry-assisted susceptibility test method. Demonstration of the workflow used. In brief, an isolate was retrieved from cryopreservation, plated onto blood agar to ensure purity, and inoculated into trypticase soya broth (TSB) overnight to simulate biological fluids. A 1 ml aliquot of this suspension was inoculated into Mueller-Hinton broth (MHB) and incubated at 37 °C to ensure bacteria were actively dividing. One aliquot was subjected to traditional microbroth dilution (MBD) susceptibility testing. Another aliquot was exposed to meropenem for 30 minutes, harvested, stained with SYTO^®^9, and then assayed with a flow cytometer. This figure is not covered by the CC-BY licence. [Credit to Life Technologies Corporation, a part of Thermo Fisher Scientific Inc. www.thermofisher.com. ^©^2016 Thermo Fisher Scientific Inc. Used under permission.] All rights reserved, used with permission.

We assembled an internationally representative panel of carbapenemase-producing *K. pneumoniae* isolates from: the Western Australian Culture Collection (WACC, PathWest Laboratory Medicine, Western Australia which included American Type Culture Collection ATCC *K. pneumoniae*: ATCC BAA1705, ATCC BAA1706 and ATCC 700603); Public Health England’s Antimicrobial Resistance and Healthcare Associated Infections (AMRHAI) Reference Unit, (London, UK); the Norwegian Public Health Institute (Oslo, Norway); and the EUCAST development Laboratory (Växjö, Sweden) (Table 1). The carbapenem MICs for all referred isolates were known, as was the mechanism of carbapenem resistance for the majority of resistant isolates (Table 2). Prior to FAST and parallel broth microdilution MIC analysis, bacteria were recovered from frozen storage in accordance with ATCC guidelines to provide a standard cell density in suspension^19^, as illustrated in Fig. 1.

**Table 1:** Assay validation with 10 *K. pneumoniae* isolates exposed to meropenem.

Isolate designation	MIC_BMD_	MIC_FAST_	Broth microdilution S/NS	FAST S/NS
ATCC 700603	≤0.25	≤0.25	S	S
	≤0.25	≤0.25	S	S
	≤0.25	≤0.25	S	S
43292	≤0.25	≤0.25	S	S
	≤0.25	≤0.25	S	S
	≤0.25	≤0.25	S	S
43358	≤0.25	≤0.25	S	S
	≤0.25	≤0.25	S	S
	≤0.25	≤0.25	S	S
18397	≤0.25	≤0.25	S	S
	≤0.25	≤0.25	S	S
	≤0.25	≤0.25	S	S
44271	0.5	0.5	S	S
	0.5	0.5	S	S
	0.5	0.5	S	S
KS1	4	4	NS	NS
	2	1	S	S
	2	2	S	S
KS11	2	1	S	S
	4	1	NS	S
	4	2	NS	S
ATCC BAA1705	16	8	NS	NS
	16	8	NS	NS
	16	8	NS	NS
K1	32	64	NS	NS
	64	64	NS	NS
	32	32	NS	NS
K14	128	256	NS	NS
	256	256	NS	NS
	256	128	NS	NS

MIC_FAST_: FAST method MIC.

MIC_BMD_: broth microdilution MIC.

S: Susceptible to meropenem, MIC ≤ 2 mg/L.

NS: Non-susceptible to meropenem, MIC > 2 mg/L.

Each isolate was exposed in biological triplicate, with each sample being acquired in technical triplicate by acoustic flow cytometry, with the MIC reported as the mode of replicates. Interpretation was according to EUCAST criteria: (S = MIC ≤ 2 mg/L; NS = MIC >2 mg/L).

**Table 2:** Meropenem susceptibilities (mg/L) for 48 *Klebsiella* isolates from diverse locations and with diverse resistance mechanisms.

Isolate	Origin	Mechanism	Referring Lab’s MIC	MIC_BMD_	MIC_FAST_	BMD S/NS	FAST S/NS
2440606	England		≤0.06	≤0.25	≤0.25	S	S
2880622	England		≤0.06	≤0.25	≤0.25	S	S
51575	Sweden	ESBL	0.064	≤0.25	≤0.25	S	S
515870	Sweden	ESBL	0.125	≤0.25	≤0.25	S	S
ATCC 700603	USA		≤0.25	≤0.25	≤0.25	S	S
ATCC BAA 1706	USA		≤0.25	≤0.25	≤0.25	S	S
3354	Australia		≤0.25	≤0.25	0.5	S	S
18397	Australia		≤0.25	≤0.25	≤0.25	S	S
43288	Australia		≤0.25	≤0.25	≤0.25	S	S
43292	Australia		≤0.25	≤0.25	≤0.25	S	S
43358	Australia		≤0.25	≤0.25	≤0.25	S	S
43854	Australia		≤0.25	≤0.25	≤0.25	S	S
A1	Norway		S	≤0.25	≤0.25	S	S
A2	Norway		S	≤0.25	≤0.25	S	S
A3	Norway		S	≤0.25	≤0.25	S	S
A4	Norway		S	≤0.25	≤0.25	S	S
A7	Norway		S	≤0.25	≤0.25	S	S
A13	Norway		S	≤0.25	≤0.25	S	S
A15	Norway		S	≤0.25	≤0.25	S	S
A16	Norway		S	≤0.25	≤0.25	S	S
2890369	England		1	≤0.25	0.5	S	S
2980639	England		1	≤0.25	0.5	S	S
10924	Sweden	KPC	4	≤0.25	0.5	S	S
3040820	England	pAmpC	≤0.06	0.5	≤0.25	S	S
270902	England		0.125	0.5	≤0.25	S	S
44271	Australia		≤0.25	0.5	0.5	S	S
K23	Australia	IMP - 4	0.5	2	0.5	S	S
500638	Sweden	pAmpC	≤0.25	4	2	NS	NS
KS1	Sri Lanka	OXA-181	1	4	2	NS	S
374	Sweden	VIM	>32	8	32	NS	NS
K16	Australia	IMP - 4	2	16	2	NS	S
ATCC BAA 1705	USA	KPC	8	16	8	NS	NS
200822	Sweden	KPC	8	16	8	NS	NS
A20	Norway	NDM-1	R	16	16	NS	NS
70708	Sweden	KPC	16	32	32	NS	NS
71076	Sweden	KPC	≥32	32	32	NS	NS
3000770	England	NDM-1	>32	32	8	NS	NS
N17	Sweden	NDM-1	≥32	32	32	NS	NS
K2	Australia	OXA-181	64	32	32	NS	NS
2880654	England	KPC	4	64	32	NS	NS
194	Sweden	VIM	≥32	64	16	NS	NS
KS17	Sri Lanka	OXA-181	≥32	64	64	NS	NS
K1	Australia	OXA-181	64	64	32	NS	NS
KS2	Sri Lanka	OXA-181	≥32	128	16	NS	NS
KS23	Sri Lanka	OXA-181	≥32	128	64	NS	NS
3080800	England	KPC	>32	256	32	NS	NS
K8	Australia	NDM-1	>256	>256	>256	NS	NS
K14	Australia	KPC	256	>256	>256	NS	NS

MIC_FAST_: FAST method MIC.

MIC_BMD_: broth microdilution MIC.

S: Susceptible to meropenem, MIC ≤ 2 mg/L.

NS: Non-susceptible to meropenem, MIC > 2 mg/L.

48 isolates were exposed to therapeutic grade meropenem and subjected to FAST in technical triplicate, with the MIC_FAST_ and MIC_BMD_ values reported as the mode of replicates. Interpretation was according to EUCAST criteria: (S = MIC ≤2 mg/L; NS = MIC >2 mg/L).

### Antimicrobial agents

Lyophilised meropenem (Ranbaxy, Haryana, India), imipenem, ertapenem and meropenem (Sigma-Aldrich, Missouri, USA) antibiotics were dissolved in sterile 0.85% saline to produce 5120 mg/L stock solutions, syringe-filtered at 0.1 µm and stored below −20 °C. FAST meropenem working stocks were made by serial 1:2 dilutions in filtered sterile Mueller-Hinton broth (MHB) to produce 1 mL aliquots ranging from 2560 mg/L to 2.5 mg/L.

### Preparation of bacteria

Fluids for bacterial preparations and acoustic flow cytometer operation were filtered at 0.1 µm prior to use to minimise particulate contamination. A 1 mL aliquot of bacterial suspension was centrifuged, washed and resuspended in 1 ml filtered Hank’s Buffered Salt Solution (HBSS) and diluted in series to 1:1000. SYTO® 9 stain working stock solution (1 µl) was added to the final dilution at a final concentration of 5 µM and incubated for 5 minutes before determination of bacterial count by flow cytometer (Attune, ThermoFisher, Eugene, OR, USA), which was used to prepare a standardised inoculum density for susceptibility testing.

An aliquot of bacterial suspension was added to 50 mL centrifuge tubes (Corning, New York) containing 9 mL of pre-warmed (37 °C) filtered MHB to produce a final density of 5 × 10^5^−1 × 10^6^ bacteria per mL. This suspension was incubated at 37 °C with shaking at 100 RPM for 30 minutes to obtain an actively dividing culture. An aliquot of antibiotic working solution from the previously described dilution series (appropriate to the concentration tested) was then added to each tube before a further 30-minute incubation with shaking, during which time a microbroth dilution (MBD) plate was prepared for overnight incubation at 37 °C^20^. One millilitre of bacterial suspension from each antibiotic concentration was harvested by centrifugation at 7800 × g for 5 minutes, washed, resuspended and diluted 1:10 in filtered HBSS in a light-impermeable microcentrifuge tube, then stained with SYTO® 9 at a final concentration of 5 µM, and incubated for 5 min before AFC sampling. Hoechst 33342 dye (NucBlue, Thermo Fisher Scientific, Eugene, OR, USA) and a SYTO® 9/propidium iodide (PI) combination (Thermo Fisher Scientific) were used in a series of replicate experiments (data not shown).

### Acoustic Flow Cytometer (AFC) operation and data analysis

The acoustic flow cytometer was calibrated at the beginning of each acquisition session in accordance with the manufacturer’s instructions (ThermoFisher Scientific). Flow cytometer settings were: Forward Scatter (FSC) voltage 3100, FSC threshold 4 × 1000 AND, blue laser 1 (BL1 – 530/30 nm) voltage 1900, BL1 threshold 1 × 1000 AND, high sensitivity, flow rate 25 µL/minute, and an acquisition volume of 125 µL. Acquisition halted after collection of 20,000 events across all gates, or after 3 minutes, with each sample acquired in technical triplicate.

Collected data were exported in the FCS 3.0 file format and analysed in Flow v10.0 (FlowJo LLC, Ashland, OR, USA) by a single user, blinded to the MIC results by broth microdilution (MIC_BMD_).

### Digital fluorescence microscopy

A 1 mL aliquot of growth from each antimicrobial concentration was harvested while conducting AFC measurements of antimicrobial-exposed bacteria, centrifuged at 7800 × g for 5 minutes, and resuspended in 10 µl of HBSS. A 0.1 µL aliquot of SYTO® 9 was added to each tube (final concentration 50 µM) and incubated for 5 minutes. A 2 µl aliquot was placed on a poly-L-lysine slide, sealed beneath a coverslip, and observed at 60x magnification by digital fluorescence microscopy. Samples were observed on the EVOS-FL digital fluorescence microscopy platform (Thermo Fisher, Eugene OR), with a representative field of view captured for each sample across the antimicrobial dilution series. High-resolution images were acquired using a Nikon Ts2R Eclipse inverted digital fluorescence microscope (Nikon, Tokyo, Japan).

### Qualitative susceptibility testing

We sought to determine whether a qualitative susceptibility test could be developed using the FAST platform. Subsequent to quantitative MIC_FAST_ determination, the flow cytometer data were re-analysed *in silico* to produce a limited sub-set for qualitative susceptibility determination. The six antibiotic dilutions (0 mg/L, 0.25 mg/L, 1 mg/L, 2 mg/L, 4 mg/L and 16 mg/L) most relevant to qualitative susceptibility assessment were used. *In silico* analysis was restricted to the first technical triplicate of each recorded sample. Gating strategies remained consistent, with the addition of a gate restricting analysis to only those events recorded in the first 60 seconds of acquisition. Isolates were defined as meropenem susceptible (S) or non-susceptible (NS) using EUCAST clinical breakpoints for Enterobacteriaceae (S ≤2 mg/L, NS >2 mg/L).

### Statistical analysis

Statistical software (Prism v 6.1, GraphPad, San Diego, CA, USA) was used to analyse both SIR categorization and quantitative MIC results. SIR results were analysed using a χ^2^ format. Clinical laboratory test performance measurements (sensitivity, specificity, positive predictive value, negative predictive value, Matthews Correlation Coefficient) were used to assess the ability of the FAST method to correctly determine carbapenem susceptibility. The correlation between MIC_BMD_ and MIC_FAST_ was analysed by calculating Spearman’s coefficient for non-parametric data. The MIC data were plotted on a log-log biaxial plot, using the microdilution results as the determinant.

### Discrepancy investigation

We examined in more detail all isolates demonstrating anomalous S/NS categories (MIC_BMD_ vs. MIC_FAST_), or MIC discrepancies outside the accepted tolerance of the microbroth dilution assay (+/− one two-fold dilution step). These isolates were subcultured once per day for three days to exclude the possibility of low prevalence contamination of cryo-preserved stocks by bacteria other than *Klebsiella* species. Any isolates displaying variable colony appearance on solid media had each observed colony morphotype sub-cultured separately, and their identity verified. We reconfirmed the molecular basis of carbapenem resistance using mechanism-specific PCR assays^5, 8^. In cases where contaminants or complex resistance mechanisms were identified, isolates were subjected to a further round of FAST following determination of identity and molecular basis of resistance.

### Subpopulation investigation

Populations observed on bi-variate flow cytometry plots that seemed to segregate into two populations across a dilution series were observed in many isolates during validation. To investigate this, we selected a demonstrative example (K16, an IMP-4 producing isolate) and subjected it to our FAST assay. We referred to this as “Day one”. One mL of the 4 mg/L meropenem exposed culture was harvested, washed in fresh HBSS to remove the presence of meropenem, and inoculated into fresh TSB to provide input for a second round of FAST on “Day two”. MIC_FAST_, population shapes, and progression to susceptibility-associated signature were compared between both experiments.

## Results

### A rapid flow-assisted susceptibility test for meropenem

Following our initial development process, we developed a new method (Fig. 1) by which susceptibility to meropenem can be assayed in *K. pneumoniae*. Using an acoustic flow cytometer to obtain optimal resolution of small particles, and a nucleic acid intercalating fluorophore to discriminate bacterial events from background debris, optimal results were achieved with SYTO^®^9. We used changes in size, shape, cytoplasmic volume and overall event numbers to predict susceptibility to meropenem in 1 hour, and MIC in 3 hours.

### Defining meropenem susceptibility by AFC

**Figure 2:**
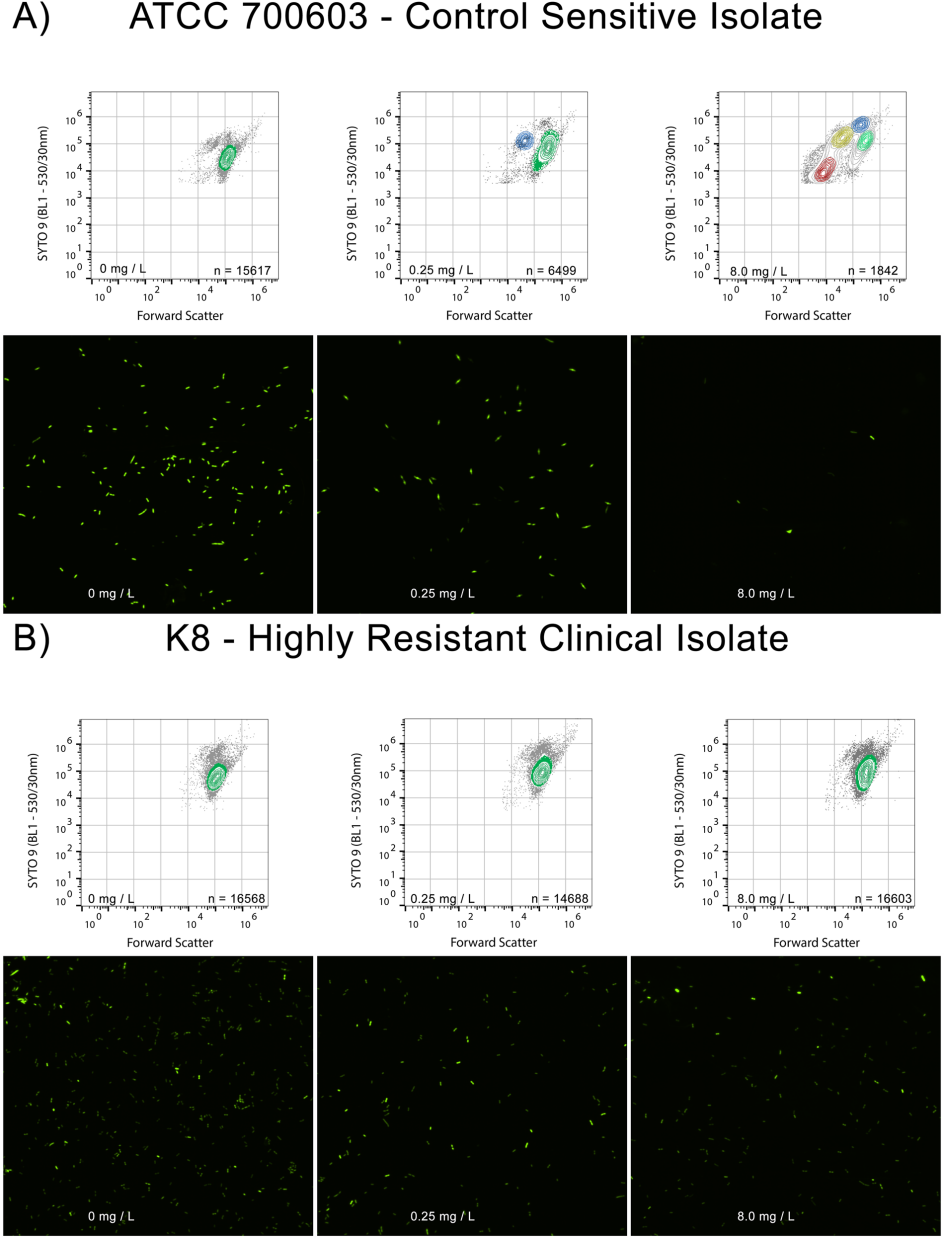
Standardised gating applied to raw data. (**A**) Collected events were gated to include only those with a SYTO®9 (BL1 - 530/30 nm) fluorescence of 10^4^ arbitrary fluorescence units or higher. Doublets were removed via a FSC-A vs FSC-H plot. Background was removed by plotting specific SYTO®9 fluorescence (BL1 – 530/30) against an unused channel (BL3 – 640 LP). (**B**) In the antibiotic unexposed sample, 10% nearest-neighbour contouring was applied, and a gate (referred to as Unexposed Cell Morphotype) was set to include all clustered events. This gate was then applied to all samples across the antibiotic dilution series.

Susceptibility to meropenem was defined by careful pairing of observed shifts in FSC and BL1 fluorescence (530/30 nm – ideal collection for SYTO®9) in bi-axial AFC plots, and observation of bacterial structures consistent with meropenem compromise by fluorescence microscopy. Exposure of actively dividing meropenem-susceptible isolates to inhibitory concentrations of the drug has been demonstrated to produce a range of cellular morphotypes; cells elongate, swell, balloon, and eventually proceed to complete cell lysis as they become compromised^21^. When microscopy and biaxial AFC plots were compared, an increased prevalence of aberrant cell morphotypes (consistent with meropenem compromise) was found to correlate with an increase in FSC, increased BL1 fluorescence, and formation of populations that contour independently on biaxial plots. In a meropenem-susceptible isolate, these changes were observed at the lowest concentration tested (Fig. 2A), whereas in an isolate with a raised meropenem MIC, these changes were not observed at concentrations below the MIC_BMD_ (Fig. 2B). When a non-susceptible isolate was exposed to concentrations approaching or exceeding its elevated MIC_BMD_, we observed forward scatter and BL1 changes associated with susceptibility. We refer here to this progressive change in morphotype approaching, and exceeding the MIC_BMD_ as the susceptibility-associated signature.

### Standardised gating strategies result in reproducible quantitative end-point report

**Figure 3:**
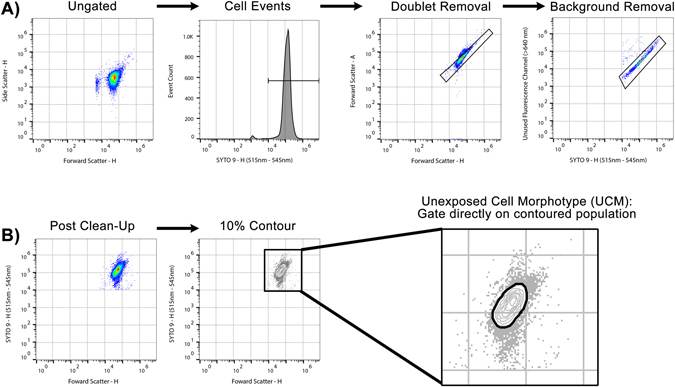
Susceptibility to meropenem can be identified by AFC by observing a susceptibility-associated signature. (**A**) Exposure of the *K. pneumoniae* susceptible type strain (ATCC 700603) increased forward scatter, SYTO®9 fluorescence, reduced overall event numbers, and formed a new contouring focus at the isolate’s MIC (0.25 mg/L). At 32 × MIC, a total of four contouring foci were observed, with an overall shift towards low forward scatter, low fluorescent debris. The progression of these features, when observed in combination, constitutes the susceptibility-associated signature. Colouring on biaxial plots indicates separate contouring foci. Fluorescence micrographs (acquired at 60x magnification) show reduced overall cell numbers and increase aberrant cell morphotypes as meropenem concentration increases. (**B**) Exposure of highly resistant clinical *K. pneumoniae* strain K8 to meropenem shows an absence of susceptibility-associated signature across clinically relevant meropenem concentrations by flow cytometer bi-axial plot, and an absence of aberrant cell morphotypes by fluorescence microscopy.

Events of interest were defined by SYTO® 9 fluorescence greater than 10^4^ arbitrary fluorescence units. Aggregate and co-incident events were excluded by rational gating on FSC-A vs FSC-H bivariate plots, and background fluorescence (auto-fluorescence and electronic noise) was minimised by rational gating on populations of interest by BL1-H vs BL3-H plot (Fig. 3A). Using the technical triplicate of the unexposed bacteria with the median BL1-H geometric mean fluorescence intensity, the auto gate tool (FlowJo) defined a gate that bounded all contoured events on a bivariate contour plot of FSC-H vs BL1-H at the 10% threshold (Fig. 3B). This gate and the events it bounded were referred to as the Unexposed Cell Morphotype (UCM), and the gate was then applied consistently to all samples across the antimicrobial agent dilution series. The absolute count of event numbers in this gate was calculated to give a comparable measure of UCM for each sample, standardised by volume (events/µL). Changes in the prevalence of morphotypes when bacteria were exposed to meropenem at concentrations approaching or exceeding the MIC_BMD_ were evident as a distinct susceptibility-associated signature. Iterative comparisons between UCM event rates/µL in antibiotic-exposed samples and the unexposed control samples were used to determine the flow-associated susceptibility test MIC (MIC_FAST_).

### Prediction of MIC_BMD_ by FC

**Figure 4:**
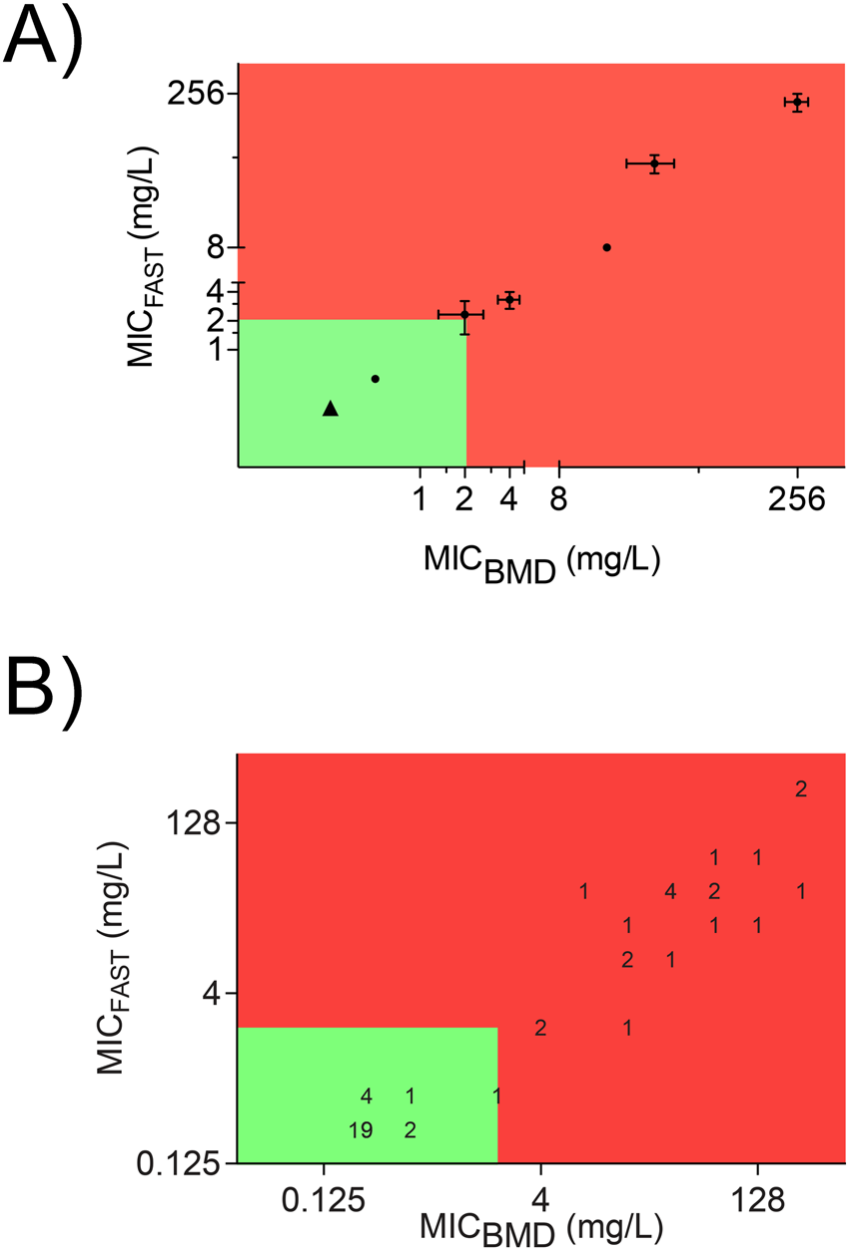
FAST accurately predicted MIC_BMD_ across our collection of *K. pneumoniae* isolates. Coloured regions represent the qualitative susceptibility (green)/non-susceptible (red) determination of (**A**) the initial 10 isolates tested (mean MIC_BMD/FAST_ and SEM), a strong positive correlation was observed (r = 0.899, p < 0.0001), represents four isolates, all with perfectly concordant MIC_BMD_,_FAST_ coordinates. (**B**) Full 48 isolate collection. Numerals indicate the number of isolates occupying the same MIC_BMD_, MIC_FAST_ coordinates. Across the full collection of isolates, a strong positive correlation was observed (Matthew’s correlation co-efficient = 0.918).

Our initial range-finding series demonstrated close correspondence between the meropenem concentrations that caused appearance of the susceptibility-associated signature in each of 10 isolates and their corresponding MIC_BMD_ values. We compared numbers of events bounded by the UCM gate per µL (UCMµ) in antibiotic-exposed samples with unexposed control samples, with a particular focus on cell numbers falling into and out of gated regions in those samples displaying the susceptibility-associated signature. We observed that the flow cytometer results accurately predicted meropenem MIC_BMD_ when a cut-off point was established as the first concentration in an antimicrobial dilution series in which two or more of the technical replicates had less than 30% of events falling into the UCM gate when compared to the unexposed control (Table 1 and Fig. 4A). We refer to this concentration as the MIC_FAST_. Paired MIC_BMD_ and MIC_FAST_ results for the entire isolate collection are shown in Table 2. There was a strong positive correlation between MIC_BMD_ and MIC_FAST_ across the entire isolate collection (Spearman r = 0. 913, p < 0.0001, sensitivity 1.00, specificity 0.90, positive predictive value 0.86, negative predictive value 1.00 and Matthew’s correlation coefficient of 0.878) (Fig. 4B). MIC_FAST_ determination required 158 minutes from actively growing culture (35 minutes of incubation, 12 minutes for manual handling, 108 minutes for data acquisition, and 3 minutes of data interpretation from a pre-prepared workspace template). Qualitative meropenem susceptibility was assessed for the entire isolate collection from the previously described data subset. Three isolates were incorrectly determined; two isolates (KS1, OXA-181, and 500638, pAmpC) were incorrectly categorised as susceptible despite being non-susceptible (MIC_FAST_ 2, MIC_BMD_ 4) however, this two-fold inter-test variation is within the accepted tolerance of the broth microdilution assay. Isolate K16 (IMP-4, MIC_BMD_ 16; MIC_FAST_ 2) was the subject of extensive further investigation. Despite these isolates, the FAST susceptible/non-susceptible threshold/interpretive criterion was highly concordant with broth microdilution-derived susceptibility (χ^2^ = 37.03, df = 1, p < 0.0001, sensitivity 1.00, specificity 0.90, positive predictive value 0.875, negative predictive value 1.00 Matthew’s correlation coefficient 0.887). Based on the conditions selected for the data set assembly, the theoretical time-to-result for this qualitative test was 54 minutes from actively dividing culture (35 minutes of incubation, 11 minutes for manual handling, six minutes for data acquisition, and two minutes of data interpretation from prepared workspace template).

### FAST can be applied to other carbapenems

To examine the applicability of the FAST method to other carbapenems, carbapenem-susceptible (ATCC 700603) and -resistant (ATCC BAA 1705) control strains of *K. pneumoniae* were exposed to analytical grade meropenem, imipenem, ertapenem, and therapeutic grade meropenem. There was no difference between S/NS categorisation between MIC_BMD_ and qualitative FAST S/NS across all tested conditions (Table 3).

**Table 3:** Control *K. pneumoniae* strains exposed to carbapenems produced concordant results between broth microdilution and FAST.

Compound Used	ATCC 700603		ATCC BAA1705	
	MIC_FAST_	MIC_BMD_	MIC_FAST_	MIC_BMD_
Therapeutic Meropenem	≤0.25	≤0.25	>16	>16
Analytical Meropenem	≤0.25	≤0.25	>16	>16
Analytical Ertapenem	≤0.25	≤0.25	>16	>16
Analytical Imipenem	1	2	>16	>16

The ATCC control carbapenem susceptible (ATCC 700603) and control carbapenem resistant (ATCC BAA1705) *K. pneumoniae* strains were exposed to three carbapenems, across the limited clinical screen configuration of concentrations. There were no differences (outside the resolution of the broth microdilution test) in MIC_FAST_ and MIC_BMD_ observed.

### MIC_BMD_ vs MIC_FAST_ discrepancy analysis

Only five of 48 (10.4%) isolates showed discrepancies between MIC_BMD_ and MIC_FAST_ that resulted in a different meropenem S/NS assessment. The first, isolate 374, was found to contain a low-prevalence *Staphylococcus aureus* contaminant. Analysing the pure *K. pneumoniae* growth produced perfect concordance between MIC_BMD_ and MIC_FAST_.

**Figure 5:**
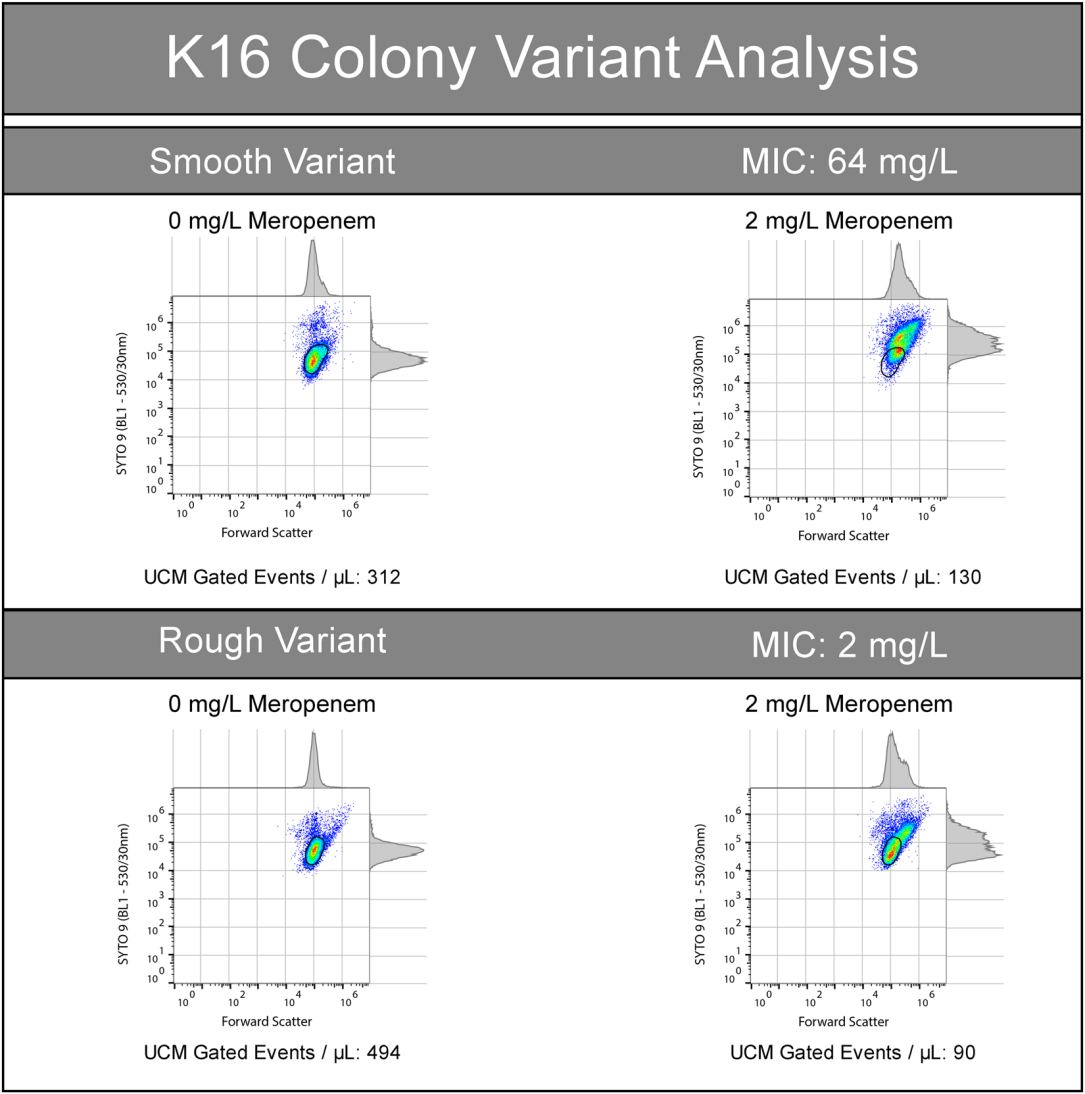
Differences in MIC_FAST_ were observed between colony variants of *K. pneumoniae* isolate K16. When subculturing IMP-4 producing *K. pneumoniae* isolate K16, a rough and smooth colony variant was observed. The smooth colony variant produced an MIC_FAST_ of 2 mg/L. The rough colony produced an MIC_FAST_ of 64 mg/L and, at 2 mg/L, was observed by AFC to contain a population consistent with a non-susceptible phenotype. Both MIC_FAST_ results were concordant with the MIC_BMD_ results.

Three of these isolates, two with an OXA-48-family enzyme and one with an IMP-4 (3000763, KS11 and K23 respectively) had MIC_BMD_ and MIC_FAST_ values within the two-fold dilution tolerance of the BMD method, but straddled the EUCAST breakpoint. This is an error of classification, not an inaccuracy of our method. The remaining isolate (K16, IMP-4) initially produced a MIC_BMD_ of 16 mg/L and a MIC_FAST_ of 2 mg/L. This was sub-cultured once again to check for purity, whereupon smooth and rough colony variants were observed. Retesting of each colony type produced an MIC_FAST_ of 2 and 64 mg/L, respectively (Fig. 5). The MIC_BMD_ (16 mg/L) and MIC_FAST_ of the rough colony variant (64 mg/L), are within the resolution of the BMD test, and result in a concordant S/NS determination.

### Identification of subpopulation and persisting populations in isolates with discrepant MIC results

**Figure 6:**
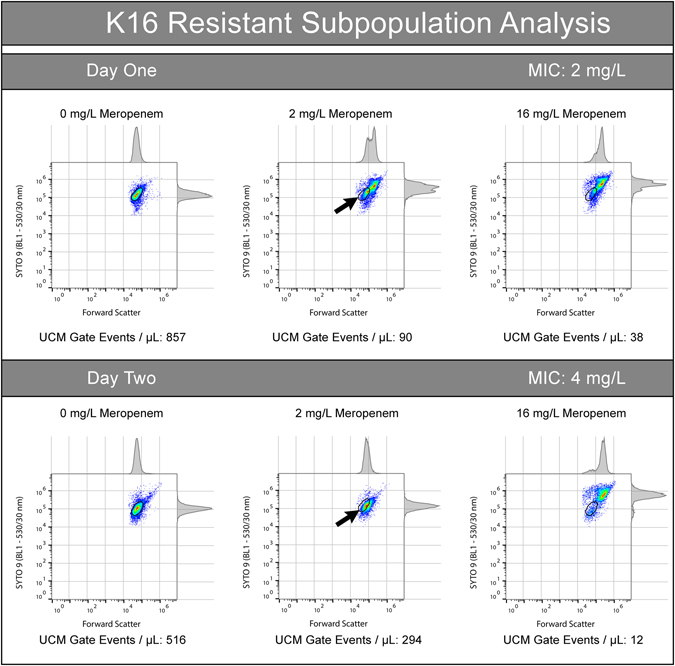
Resistant sub-populations were observed in *K. pneumoniae* isolate K16 across two days of selective passage and FAST: Day One - IMP-4 producing *K. pneumoniae* isolate K16 was found, at 2 mg/L meropenem, to contain a minority population of cells with a phenotype consistent with unexposed cells (remaining within the Unexposed Cell Morphotype gate - indicated by arrow). This subpopulation persisted, at a diminished frequency, at 16 mg/L meropenem while the majority of cells display a compromised phenotype (shifted outside the gate). Day 2 – The 2 mg/L culture of K16 from Day 1 was subcultured and subjected to FAST on the following day. The isolate displayed an increased MIC (4 mg/L), delayed progression to the emergence susceptibility-associated signature, with most events consistent with a non-susceptible phenotype at 2 mg/L. Most events at 16 mg/L were consistent with a susceptible phenotype, however a small subpopulation remained inside the Unexposed Cell Morphotype gate.

On Day 1, isolate K16, classified as susceptible by MIC_FAST_, was found to contain a population of bacterial events consistent with unexposed cell morphotypes that persisted until 16 mg/L (Fig. 6 - Day 1). On Day 2 the bacterial population characteristics exhibited a different progression towards the susceptibility-associated signature across the same meropenem dilution range. Bacterial cells had a much higher forward scatter, without an associated BL1 increase on Day 2, and starting at 4 mg/L a subpopulation of cells again became evident (Figure Day 2). Subpopulations such as these have been observed across approximately one third of isolates assayed in our collection (n = 17).

## Discussion

Antimicrobial susceptibility profiling of carbapenem-resistant *K. pneumoniae* by acoustic flow cytometer predicted both quantitative (MIC) and qualitative (susceptible/non-susceptible) carbapenem susceptibility. While flow cytometry has been used for antimicrobial susceptibility testing before^11–18^, our FAST assay is the first reported description of a validated method to generate a clinically-relevant quantitative end-point. Furthermore, our rapid phenotypic determination of antimicrobial susceptibility accurately predicts the qualitative result, and is therefore a significant step towards alignment of laboratory testing with clinical decision timelines. Broth microdilution is too labour-intensive for use in most clinical laboratories, which favour other methods of susceptibility determination. We present performance statistics for our qualitative susceptibility test but to demonstrate the power of single-cell level analysis rather than to expect immediate adoption of this assay in the clinical laboratory. To the prescribing physician, rapid qualitative susceptibility represents an ability to align the decision-making process of antibiotic prescribing to the best-practice ideals of effective anti-microbial stewardship^7^.

The FAST method is suitable for application as a rapid method to determine carbapenem resistance phenotype on the grounds of a strong correlation between MIC_BMD_ and MIC_FAST_. MIC_FAST_ follows a pre-determined heuristic to generate quantitative results, rather than relying on potentially user-biased subjective end-points. Our use of workspace templates allowed replication of results by non-specialists after minimal instruction by a skilled operator using a proprietary software package (FlowJo). Furthermore, any flow cytometry software capable of generating a contouring output should be suitable. The FAST assay is underpinned by the reproducible flow cytometry model of a complex series of physiological interactions we established. Forward Scatter (FSC) is often used a surrogate for particle size, but this oversimplifies the dynamic behaviour of non-spherical particles^17^. There is much more information in this single measurement than the size and orientation of a particle passing through the flow cytometer. For example, changes in granularity and autofluorescence profiles also alter the absolute numbers of photons reaching the FSC detector, and in similar manner, photons absorbed and emitted by fluorescence signals can alter FSC measurements^17, 18^. Our choice of fluorescent dye (SYTO^®^ 9) ensured that measurements in the BL1 channel (530/30 nm) contained information on DNA content, cytoplasmic volume and autofluorescence. Observed staining intensity profiles from a rigorously controlled experimental method offer additional insight into physiological properties such as membrane permeability and dye molecule efflux^14^. Isolates with the osmoporin *ompK36* third eyelet insertion mutation (ins AA 134-135 GD^5, 6^) displayed a reduced BL1 intensity. This porin mutation excludes positively charged compounds such as SYTO®9^22^ and has been shown to correlate with high-level meropenem resistance^23, 24^. The consistency of our observations across a collection of isolates from such diverse geographic origins and resistance mechanisms supports a conserved bacterial physiology.

The physiological response we detected by the FAST method after antimicrobial exposure resembles the range of carbapenem-induced morphotypes described previously^21^. Arrested cell division after inhibition of penicillin-binding proteins^25, 26^ leads to an overall increase in cellular DNA and increases the DNA-bound SYTO® detectable in BL1. The overall decrease in cell numbers by fluorescence microscopy and flow cytometry, and the corresponding increase in flow cytometer event populations with low forward scatter and varied BL1, is likely to reflect mixed cell debris from bacterial cell lysis during antimicrobial exposure. The broth microdilution MIC method relies on a subjective end-point and requires extended incubation^27^, allowing persistence of resistant sub-populations after inhibition of the susceptible majority of bacteria^28–30^. The FAST method measures the resistance phenotype of all bacterial cells in each aliquot, and adds to the evidence that carbapenem-resistant Enterobacteriaceae are phenotypically heterogeneous^28–30^. We postulate that broth microdilution over-simplifies the test method and overestimates the dose required to demonstrate antimicrobial efficacy. Highly resistant bacterial sub-populations have been implicated in failed meropenem monotherapy before^28, 31^. These bacteria may respond to meropenem combination therapy provided sufficient breakthrough growth has not occurred^28, 31^. Identification of these features of bacterial susceptibility in a shorter time could become the basis of more timely antimicrobial treatment guidance^28, 29, 31^. While our method eliminates the necessity of the secondary culture step required for either broth microdilution or other growth-dependent quantitative susceptibility determination^27^, further advances are needed to purify bacteria directly from patient samples so that laboratory results are available to the physician within a shorter time frame, particularly for patients with sepsis and other severe infections.

Discrepancies of ≥2 two-fold dilutions were observed between MIC_BMD_ and MIC_FAST_ for pAmpC- and IMP-4-producing isolates. These types of resistance cause inducible meropenem resistance^30, 32^. Induction of meropenem-resistant pAmpC-producing *K. pneumoniae* has been demonstrated after accumulation of transpeptidation by-products in the cytosol^32–35^. Selection of low-prevalence sub-populations with constitutive AmpC can also lead to rapid, time-dependent shifts in the overall resistance phenotype^33, 34^. Induction of expression does not occur within 30 minutes of antimicrobial exposure and may therefore contribute to discrepancies between MIC_BMD_ and MIC_FAST_. In the case of IMP-4-producing isolates, high-level induced meropenem resistance is thought to be caused by intrinsically-resistant sub-populations^30^. The presence of persistent bacterial populations at higher meropenem concentrations in the UCM gate indicates a sub-population of inducible IMP-4-mediated meropenem resistant cells. Identification of inducible resistance is a challenge with any antimicrobial susceptibility test, but determination of the result shortly after the start of antimicrobial exposure should reduce the complex effects of prolonged antimicrobial exposure and improve the accuracy of test endpoints.

Our assay for MIC_FAST_,has potential research applications such as resistant phenotype surveillance^5–7^, determining the impact of altered environmental/chemical conditions on resistance phenotype^24–38^, and modelling the interactions between mixed bacterial populations following antimicrobial insult. A potential use for this assay is its incorporation in a suite of orthogonal analyses that combine genomic and phenotypic investigations to assess the physiological features of a particular resistance phenotype^24–26, 30–36^.

The precision of our method for determining quantitative and qualitative susceptibility to meropenem in *Klebsiella* species compares favourably with the current international standard, while returning results in 1 (qualitative) to 3 (quantitative) hours after receipt of primary culture – in most cases a full 24 hours earlier than current standard practice. Transition from subjectively interpreted end-points to objectively-generated, single bacterial cell analysis can improve the resolution of an antimicrobial susceptibility test, without sacrificing either precision or specificity.
