# Author Correction: Rapid susceptibility profiling of carbapenem-resistant *Klebsiella pneumoniae*

**DOI:** 10.1038/s41598-018-25216-y

**Published:** 2018-04-24

**Authors:** K. T. Mulroney, J. M. Hall, X. Huang, E. Turnbull, N. M. Bzdyl, A. Chakera, U. Naseer, E. M. Corea, M. J. Ellington, K. L. Hopkins, A. L. Wester, O. Ekelund, N. Woodford, T. J. J. Inglis

**Affiliations:** 1 Harry Perkins Institute of Medical Research, School of Medicine, Faculty of Health and Medical Sciences, the University of Western Australia, Nedlands, Western Australia Australia; 2 Marshall Centre, School of Biomedical Sciences, Faculty of Health and Medical Sciences, the University of Western Australia, Nedlands, Western Australia Australia; 3 Department of Microbiology, PathWest Laboratory Medicine, WA Nedlands, Australia; 4 Norwegian Institute of Public Health, Oslo, Norway; 5 Department of Microbiology, University of Colombo, Kynsey Road, Colombo, Sri Lanka; 6 Antimicrobial Resistance and Healthcare Associated Infections (AMRHAI) Reference Unit, National Infection Service, Public Health England, London, NW9 5EQ UK; 7 Department of Clinical Microbiology and EUCAST Development Laboratory, Region Kronoberg, Växjö, Sweden; 8 Division of Pathology and Laboratory Medicine, School of Medicine, Faculty of Health and Medical Sciences, the University of Western Australia, Nedlands, Western Australia Australia

Correction to: *Scientific Reports* 10.1038/s41598-017-02009-3, published online 15 May 2017

In this Article, the images for Figures 2 and 3 were inadvertently switched. The correct Figures 2 and 3 appear below with their accompanying figure legends as Figures 1 and 2, respectively. The Figure legends are correct.

**Figure 1:**
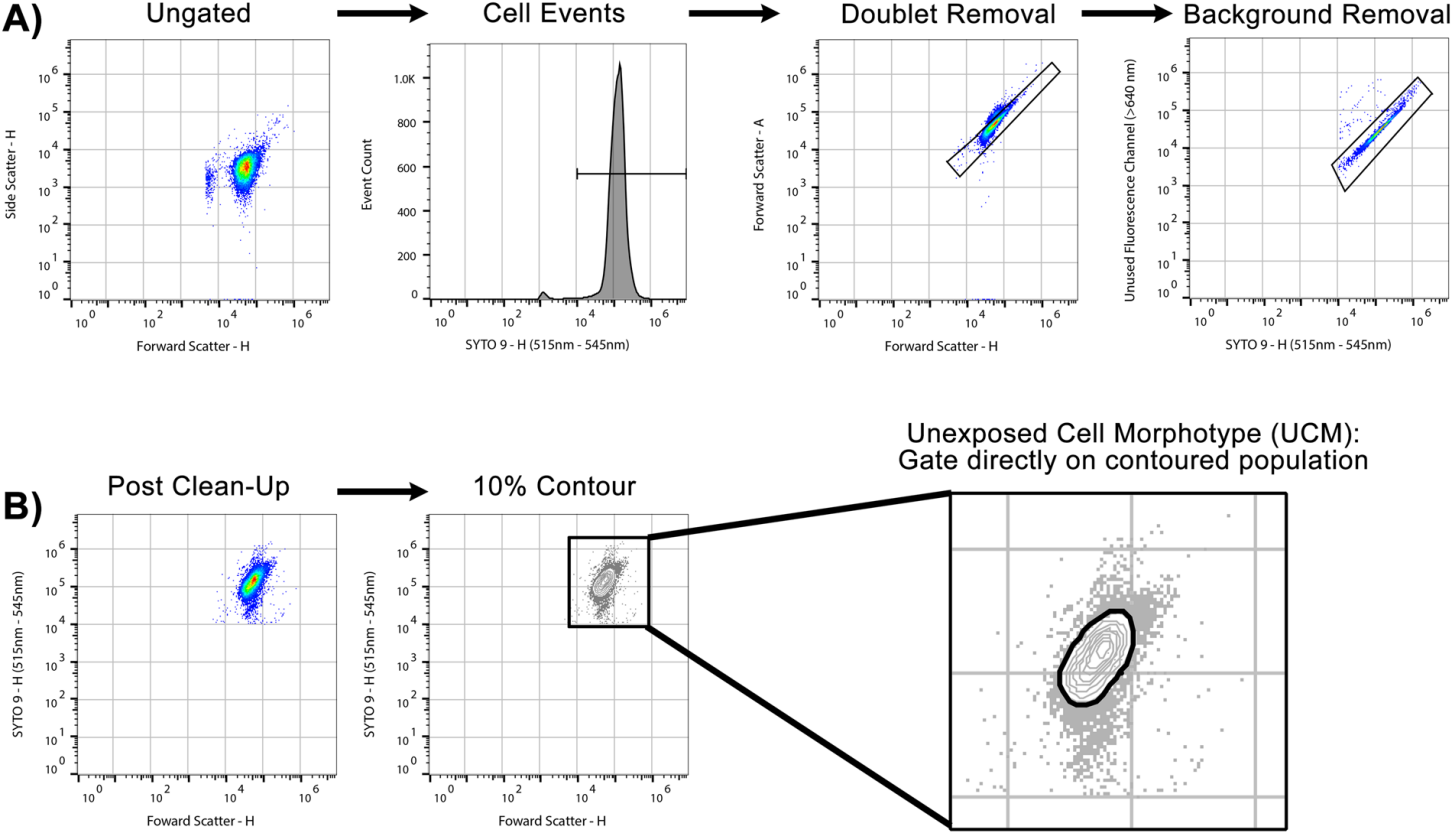
Standardised gating applied to raw data. (**A**) Collected events were gated to include only those with a SYTO®9 (BL1 - 530/30 nm) fluorescence of 10^4^ arbitrary fluorescence units or higher. Doublets were removed via a FSC-A vs FSC-H plot. Background was removed by plotting specific SYTO®9 fluorescence (BL1 – 530/30) against an unused channel (BL3 – 640 LP). (**B**) In the antibiotic unexposed sample, 10% nearest-neighbour contouring was applied, and a gate (referred to as Unexposed Cell Morphotype) was set to include all clustered events. This gate was then applied to all samples across the antibiotic dilution series.

**Figure 2:**
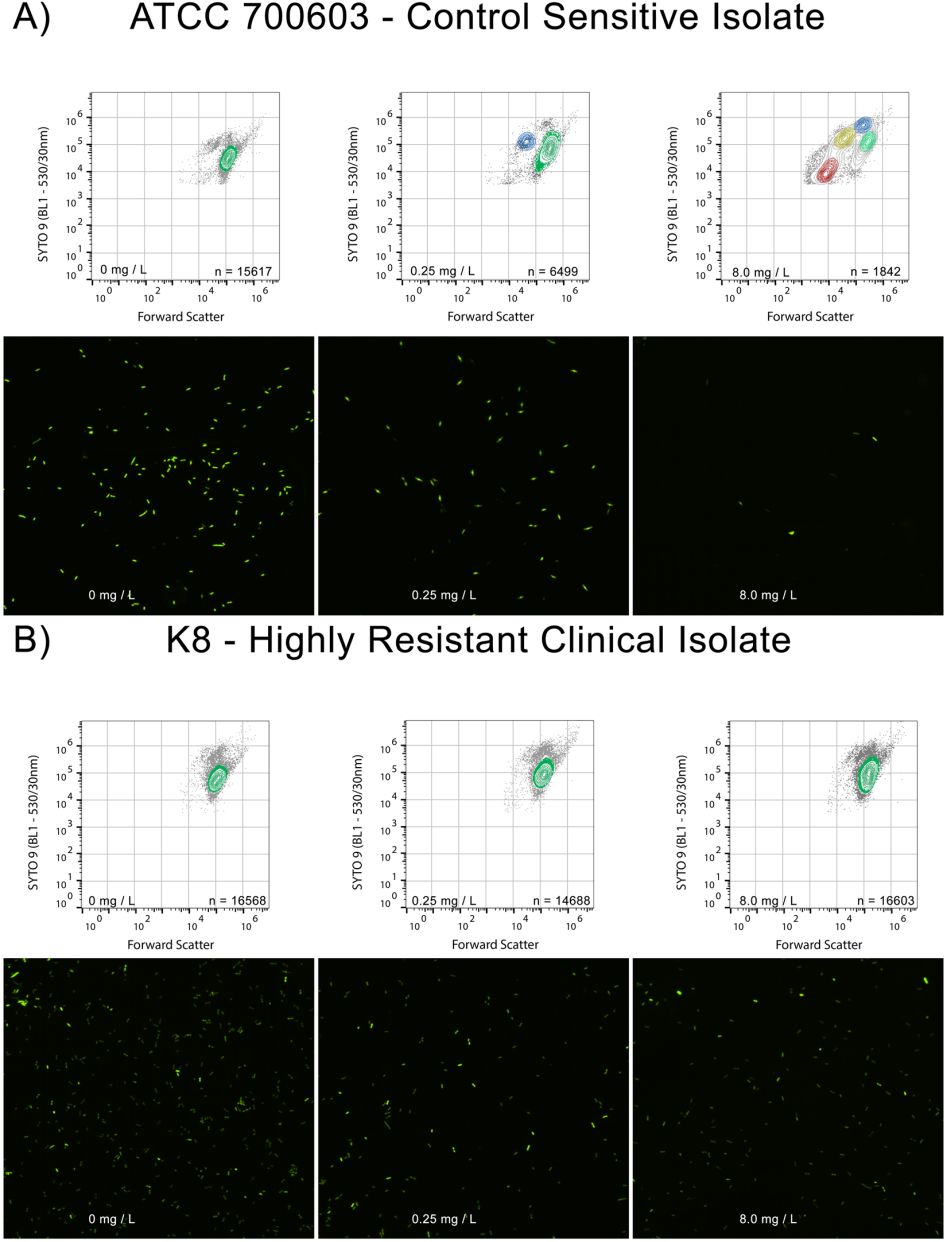
Susceptibility to meropenem can be identified by AFC by observing a susceptibility-associated signature. (**A**) Exposure of the *K. pneumoniae* susceptible type strain (ATCC 700603) increased forward scatter, SYTO®9 fluorescence, reduced overall event numbers, and formed a new contouring focus at the isolate’s MIC (0.25 mg/L). At 32 × MIC, a total of four contouring foci were observed, with an overall shift towards low forward scatter, low fluorescent debris. The progression of these features, when observed in combination, constitutes the susceptibility-associated signature. Colouring on biaxial plots indicates separate contouring foci. Fluorescence micrographs (acquired at 60x magnification) show reduced overall cell numbers and increase aberrant cell morphotypes as meropenem concentration increases. (**B**) Exposure of highly resistant clinical *K. pneumoniae* strain K8 to meropenem shows an absence of susceptibility-associated signature across clinically relevant meropenem concentrations by flow cytometer bi-axial plot, and an absence of aberrant cell morphotypes by fluorescence microscopy.

